# Synthesis, crystal structure and Hirshfeld surface analysis of phenyl­methanaminium 2-oxo-2*H*-chromene-3-carboxyl­ate

**DOI:** 10.1107/S2056989026003403

**Published:** 2026-04-10

**Authors:** M. Sunithakumari, M.U. Gagan, V. Dwarakanath, H.T. Srinivasa, H. C. Devarajegowda, B. S. Palakshamurthy

**Affiliations:** a Department of Physics Yuvaraja's College University of Mysore,Mysore 570005 Karnataka India; b Department of Biotechnology, UCS Tumkur University Tumkur Karnataka-572103 India; c Raman Research Institute, C V Raman Avenue Sadashivanagar Bangalore-560080 Karnataka India; d Department of PG Studies and Research in Physics Albert Einstein Block UCS Tumkur University, Tumkur Karnataka-572103 India; Katholieke Universiteit Leuven, Belgium

**Keywords:** crystal structure, co-crystals, Hirshfeld surface, 2-oxo-2*H*-chromene, benzyl amine

## Abstract

The title salt, C_7_H_10_N^+^·C_10_H_4_O^−^, formed between 2-oxo-2*H*-chromene-3-carb­oxy­lic acid and benzyl­amine crystallizes in the triclinic space group *P*1. Proton transfer from 2-oxo-2*H*-chromene-3-carb­oxy­lic acid to the NH_2_ group of benzyl­amine results in a N—H⋯(O,O) hydrogen bond between cation and the carboxylate group of the anion. The 2-oxo-2*H*-chromene moiety is almost planar with a dihedral angle between the two fused rings of 1.48 (11)°. The dihedral angle between the rings of the anion and cation is 29.49 (10)°. In the crystal, N—H⋯O hydrogen-bonding inter­actions generate an *R*_4_^4^(12) synthon parallel to the *ac* plane. The mol­ecules are linked by further C—H⋯π inter­actions, which consolidate the packing. In addition, π–π stacking inter­actions are observed with centroid-to-centroid distances of 3.5832 (14) and 3.8167 (15) Å. The two-dimensional fingerprint plots indicates that the most important contributions to the crystal packing are from H⋯H (39.7%), H⋯O/O⋯H (30.6%) and H⋯C/C⋯H (20.9%) contacts. The anti­bacterial activity, with MIC values of 30 µg ml^−1^ against *S. aureus* and 25 µg ml^−1^ against *E. coli*. The lower MIC against *E. coli* suggests that the compound is more effective against Gram-negative bacteria than Gram-positive bacteria.

## Abstract 2

The title salt, C_7_H_10_N^+^·C_10_H_4_O^−^, crystallizes in the triclinic space group *P*1 with *Z* = 2. The structure of the 1:1 co-crystal formed between 2-oxo-2*H*-chromene-3-carb­oxy­lic acid and benzyl­amine features an N—H⋯O hydrogen bond. A Hirshfeld surface analysis, anti­bacterial studies and inter­molecular inter­action energies within the crystal structure are reported.

## 1. Chemical context

Chromene and coumarin derivatives possess considerable pharmacological relevance. 2*H*-Chromene oxime derivatives exhibit anti­proliferative activity against A549, MCF-7 and MDA-MB-231 cell lines (Bandaru *et al.*, 2025 ▸), while coumarin-3-carboxamides demonstrate both anti­bacterial and anti­cancer properties (Phutdhawong *et al.*, 2021 ▸). Similarly, 2-oxo-2*H*-chromene-3-carboxyl­ates show cytotoxicity toward HepG2, HeLa and HCT116 tumour cell lines (Ji *et al.*, 2021 ▸), and related chromene-3-carboxyl­ates display activity against both Gram-positive and Gram-negative bacteria (Venugopala *et al.*, 2013 ▸). Coumarins are also known inhibitors of cyclo­oxygenase and lipoxygenase pathways (Stefanachi *et al.*, 2018 ▸). Furthermore, chromene derivatives constitute validated pharmacophores in anti­coagulant therapy; clinically used agents inhibit vitamin K epoxide reductase, thereby suppressing vitamin K-dependent clotting factors (Ansell, 2008 ▸).

Beyond pharmaceutical applications, functionalized phenyl­methanaminium iodide salts have been investigated as surface-passivation agents in perovskite solar cells, improving device efficiency and operational stability (Yasa *et al.*, 2025 ▸). Phenyl­methanaminium (benzyl­ammonium) derivatives additionally exhibit diverse biological activities, including anti­tumor (Kemnitzer *et al.*, 2004 ▸), anti­bacterial (Ganapathi *et al.*, 2025 ▸) and anti-inflammatory effects (Cacabelos *et al.*, 2024 ▸). Collectively, these findings highlight the structural versatility and biological significance of chromene and phenyl­methanaminium frameworks and support their continued investigation in crystal engineering and pharmaceutical materials research. s part of our studies in this area, we now report the synthesis and structure of the title salt C_7_H_10_N^+^·C_10_H_4_O^−^, (I). 
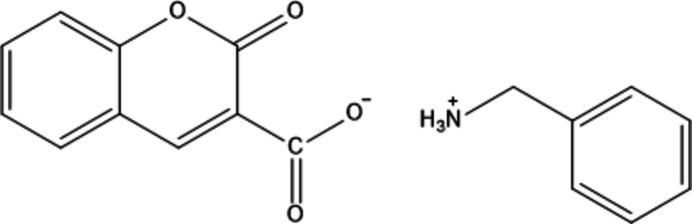


## 2. Structural commentary

**Figure 1:**
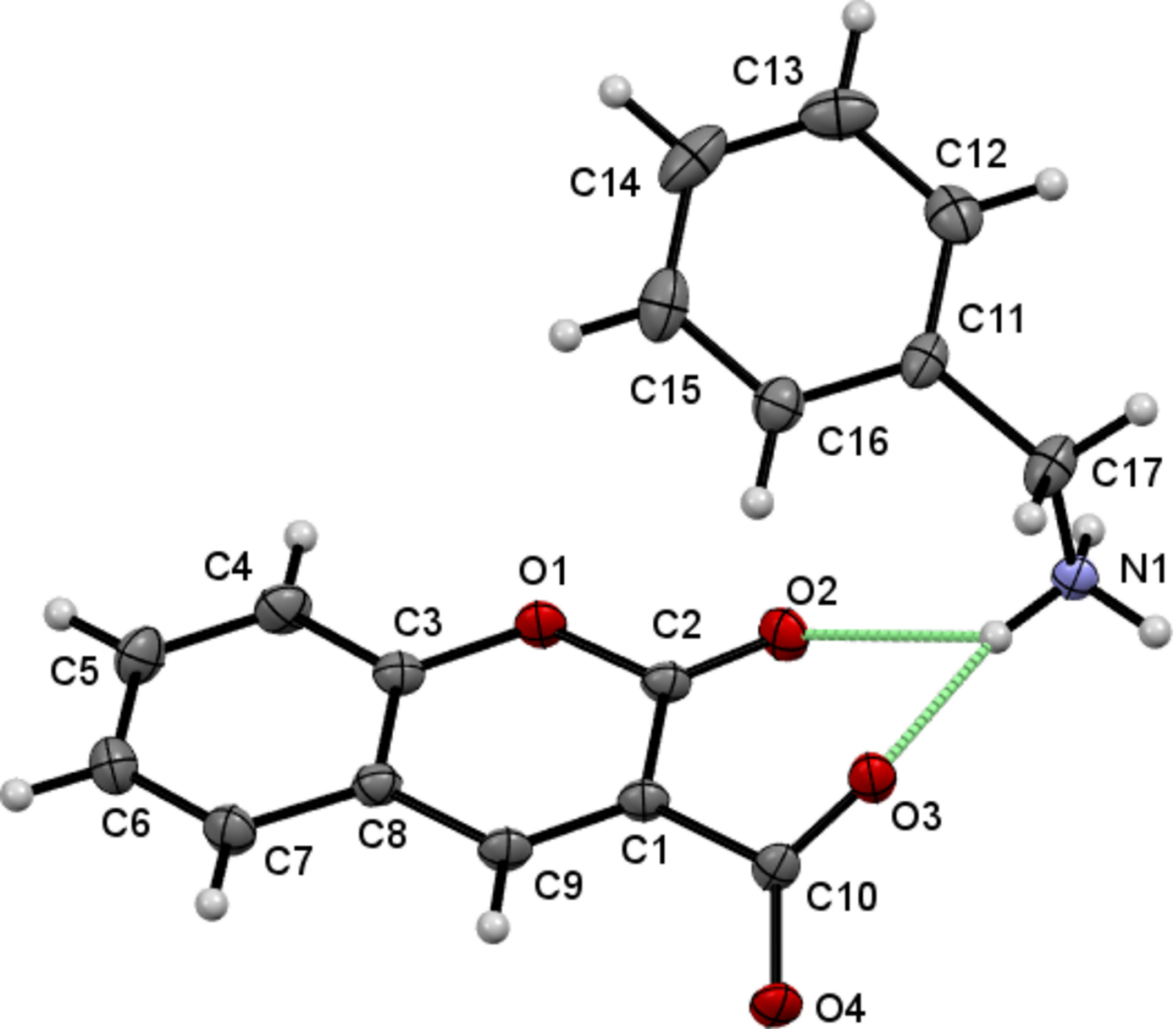
The title salt with atom-numbering scheme and 50% probability ellipsoids. Dashed lines represent hydrogen bonds.

The mol­ecular structure of (I) is shown in Fig. 1 ▸. The dihedral angle between the rings of the almost planar (r.m.s. deviation = 0.015 Å) fused ten-membered 2-oxo-2*H*-chromene moiety is 1.48 (11)° whereas the dihedral angle between 2-oxo-2*H*-chromene and the aromatic ring of benzyl­amine is 29.49 (10)° for the species in the asymmetric unit. Proton transfer from the 2-oxo-2*H*-chromene-3-carb­oxy­lic acid to the NH_2_ substituent of ethyl­benzene lead to the formation of the title salt with a strong N1—H1*A*⋯O3 hydrogen bond (Table 1 ▸). The torsion angles C2—C1—C10—O3 and N1—C17—C11—C16 are 44.9 (3) and 78.0 (3)°, respectively. The angle made by the atoms C11—C17—N1 is 113.3 (2)°.

## 3. Supra­molecular features

**Figure 2:**
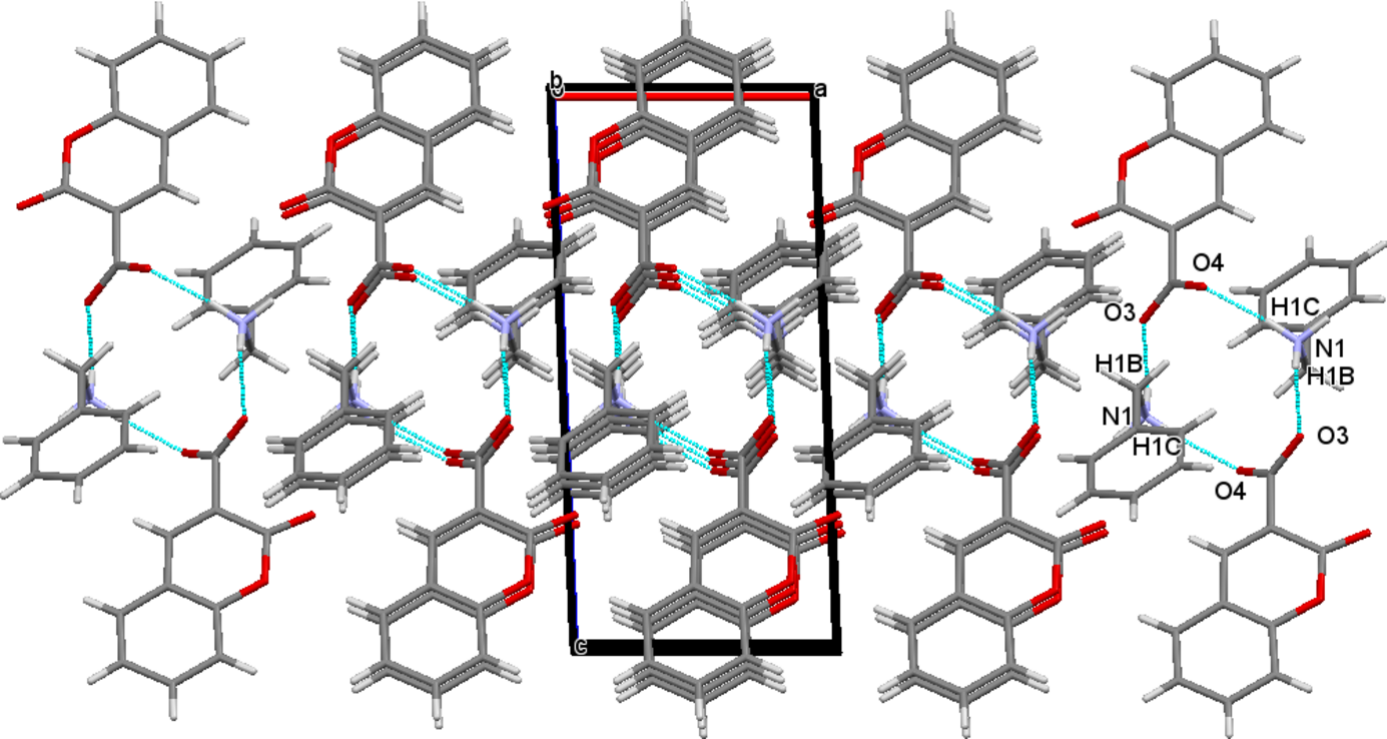
Packing diagram with N—H⋯O hydrogen bonds shown as blue dashed lines.

**Figure 3:**
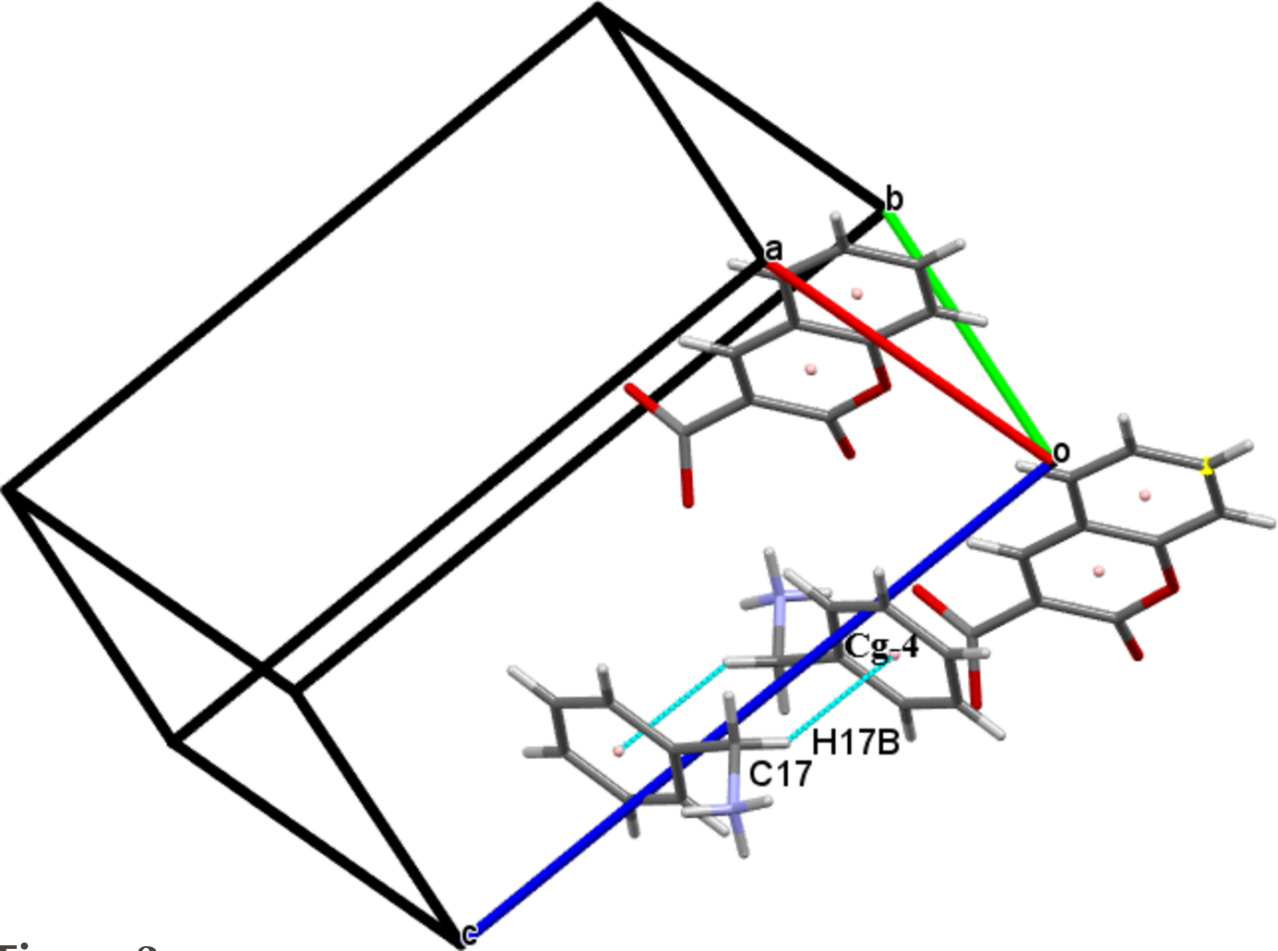
Partial packing diagram with C—H⋯π inter­actions shown as blue dashed lines.

**Figure 4:**
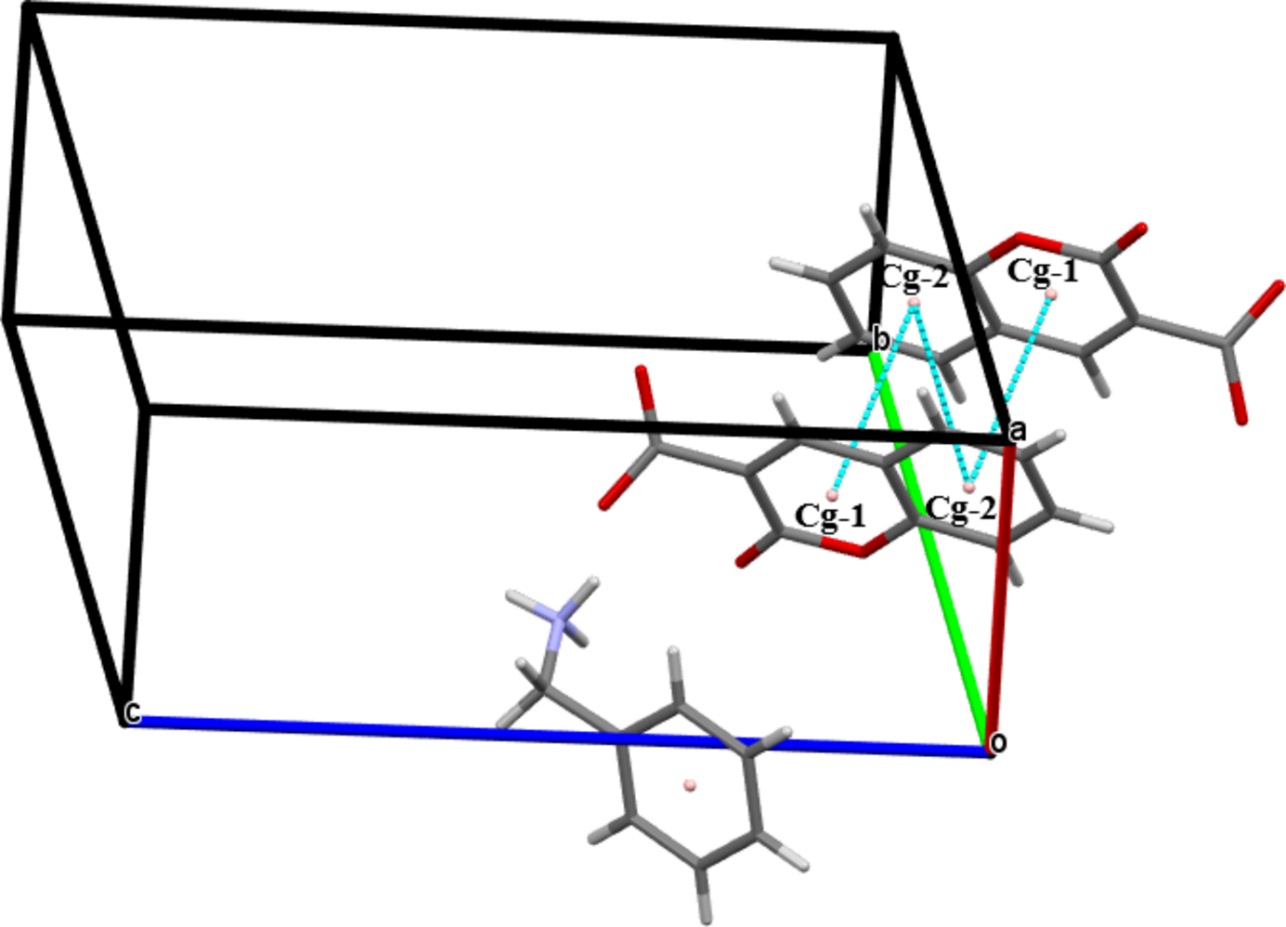
The mol­ecular packing of (I) with π⋯π stacking inter­actions shown as blue dashed lines.

In the crystal, the ions are linked by weak N1—H1*A*⋯O2 and stronger N1—H1*A*⋯O3 hydrogen bonds (Table 1 ▸). A set of 2-oxo-2*H*-chromene-3-carboxyl­ate phenyl­methanaminium mol­ecules generate a layered two-dimensional supra­molecular architecture propagating in the *ac* plane as shown in Fig. 2 ▸. The tetramer (two cations and two anions) of mol­ecules generate an 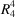
(12) synthon. Two nitro­gen donor atoms, one from each phenyl­methanaminium cation and four oxygen acceptor atoms, two from each 2-oxo-2*H*-chromene-3-carboxyl­ate cation, are involved in the synthon. The mol­ecules are linked by C17—H17*B*⋯*Cg*4 inter­actions (*Cg*4 is the centroid of the C11–C16 ring; Fig. 3 ▸). The crystal packing is further consolidated by π–π stacking inter­actions with centroid-to-centroid distances *Cg*1⋯*Cg*2^i^ = 3.5832 (14) Å and *Cg*2⋯*Cg*2^i^ = 3.8167 (15) Å [slippage = 1.675 Å; symmetry code: (i) 1 − *x*, 1 − *y*, −*z*′; *Cg*1 and *Cg*2 are the centroids of the O1/C2/C1/C9/C8/C3 and C3–C8 rings, respectively; Fig. 4 ▸].

## 4. Database survey

A search of the Cambridge Structural Database (CSD, version 5.42, November 2020 update; Groom *et al.*, 2016 ▸) for compounds containing a 2-oxo-2*H*-chromene-3-carboxyl­ate moiety yielded over thirty entries. Among these, butane-1,4-di­ammonium bis­(2-oxo-2*H*-chromene-3-carboxyl­ate) CSD (refcode GEPLUK; Das *et al.*, 2012 ▸), 4-(3,4-di­chloro­phen­yl)-*N*-methyl-1,2,3,4-tetra­hydro­naphthalen-1-aminium 2-oxo-2*H*-chromene-3-carboxyl­ate (VAHHEU; Escudero *et al.*, 2016 ▸), and 2,6-di­amino­pyridinium 2-oxo-2*H*-chromene-3-carboxyl­ate (VAXRIX; Yan *et al.*, 2012 ▸) bear similar 2-oxo-2*H*-chromene substituents and exhibit a dihedral angle between both rings of the ten-membered 2-oxo-2*H*-chromene moiety of 1.86, 1.96, and 0.44°, respectively, comparable to the title compound [1.48 (11)°].

Furthermore, a search for phenyl­methanaminium fragments also yielded over thirty entries. Among these, three structures are comparable to the title salt: phenyl­meth­an­aminium naphthalene-2-sulfonate (DOXLEK01; Chak­raborty *et al.*, 2020 ▸), dodeca­kis­(phenyl­methanaminium) tris­(benzene-1,2,4,5-tetra­carboxyl­ate) octa­hydrate (EZO­CUU; Ye *et al.*, 2021 ▸), and phenyl­methanaminium 4-(2,4,6-triiso­propyl­benzo­yl) benzoate (CARGIM01; Bąkowicz *et al.*, 2014 ▸). In these structures, the C(ring)—C—N angle is 112.0, 114.8, and 113.5°, comparable to 113.3 (2)° for the title salt.

## 5. Hirshfeld surface analysis

**Figure 5:**
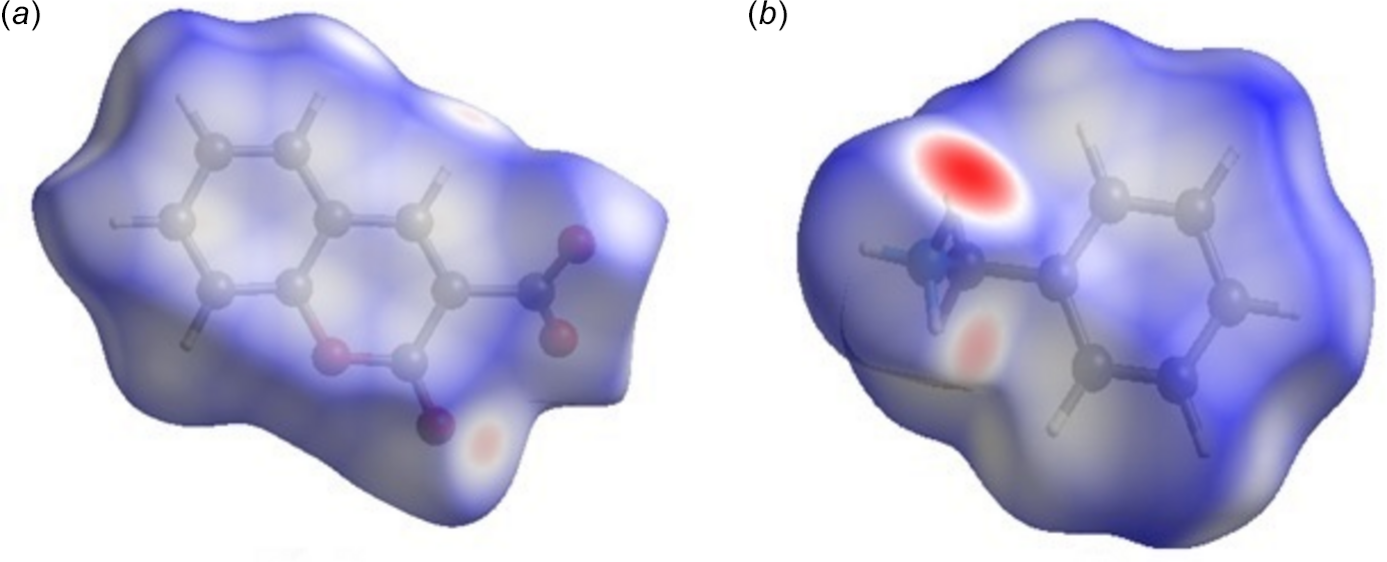
View of the three-dimensional Hirshfeld surface of the 2-oxo-2*H*-chromene-3-carboxyl­ate moiety (*a*) and the phenyl­methanaminium fragment (*b*) plotted over *d*_norm_.

**Figure 6:**
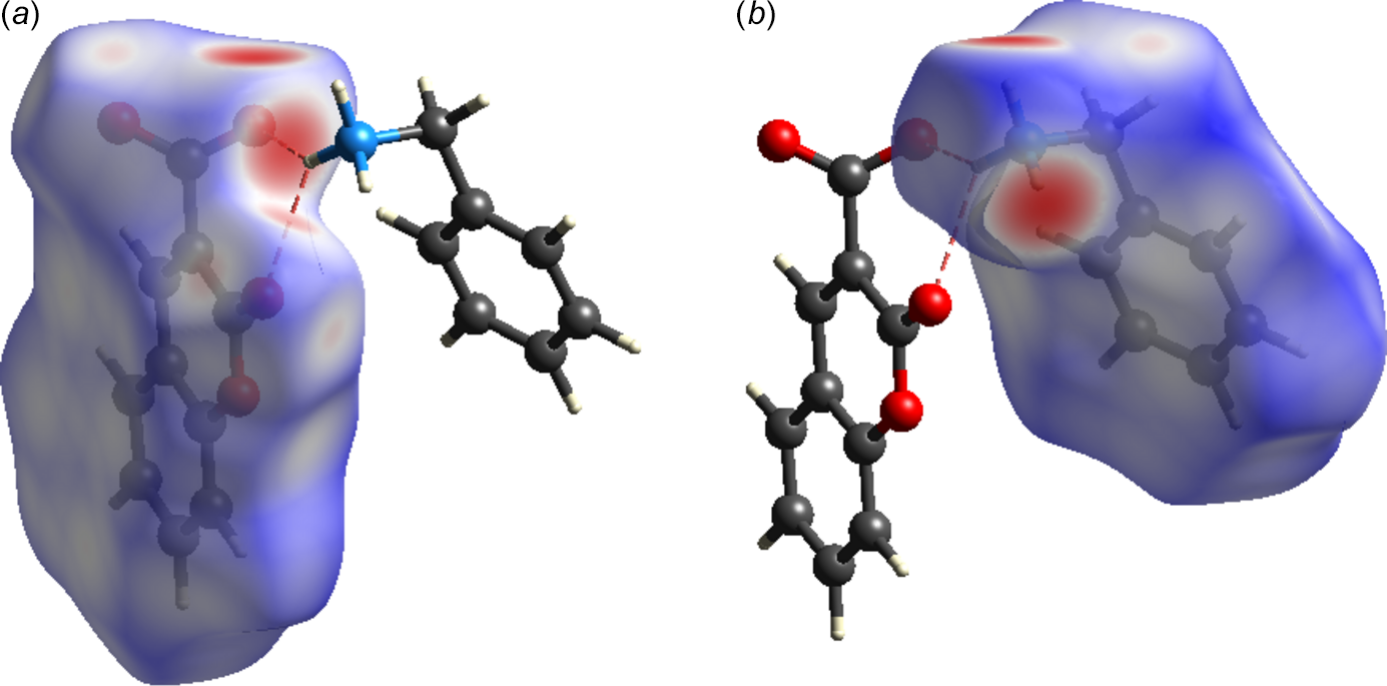
Hirshfeld surface of (I) plotted over *d*_norm_ with the N—H⋯O inter­actions near the red spots shown for the 2-oxo-2*H*-chromene-3-carboxyl­ate moiety (*a*) and the phenyl­methanaminium fragment (*b*)

**Figure 7:**
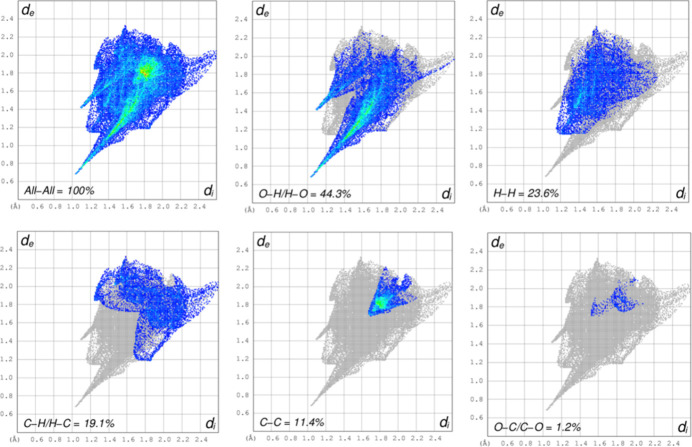
The two-dimensional fingerprint plots for the 2-oxo-2*H*-chromene-3-carboxyl­ate moiety, showing all inter­actions, and delineated into O⋯H/H⋯O (44.3%), H⋯H (23.6%), H⋯C/C⋯H (19.1%), C⋯C (11.4%), and O⋯C/C⋯O (1.2%) contacts.

**Figure 8:**
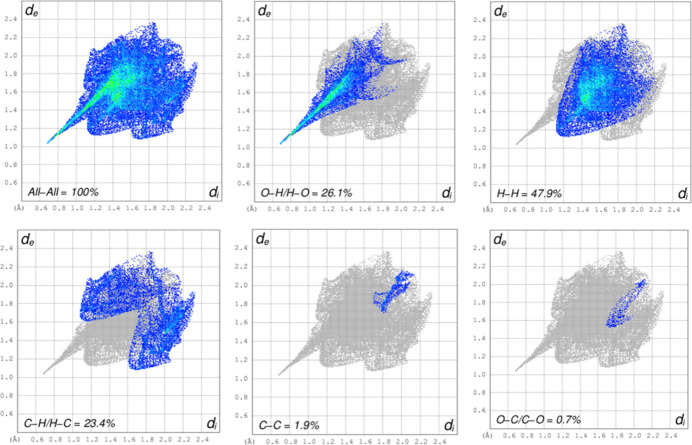
The two-dimensional fingerprint plots for the phenyl­methanaminium moiety, showing all inter­actions, and delineated into O⋯H/H⋯O (26.1%), H⋯H (47.9%), H⋯C/C⋯H (23.4%), C⋯C (1.9%), and O⋯C/C⋯O (0.7%) contacts.

**Figure 9:**
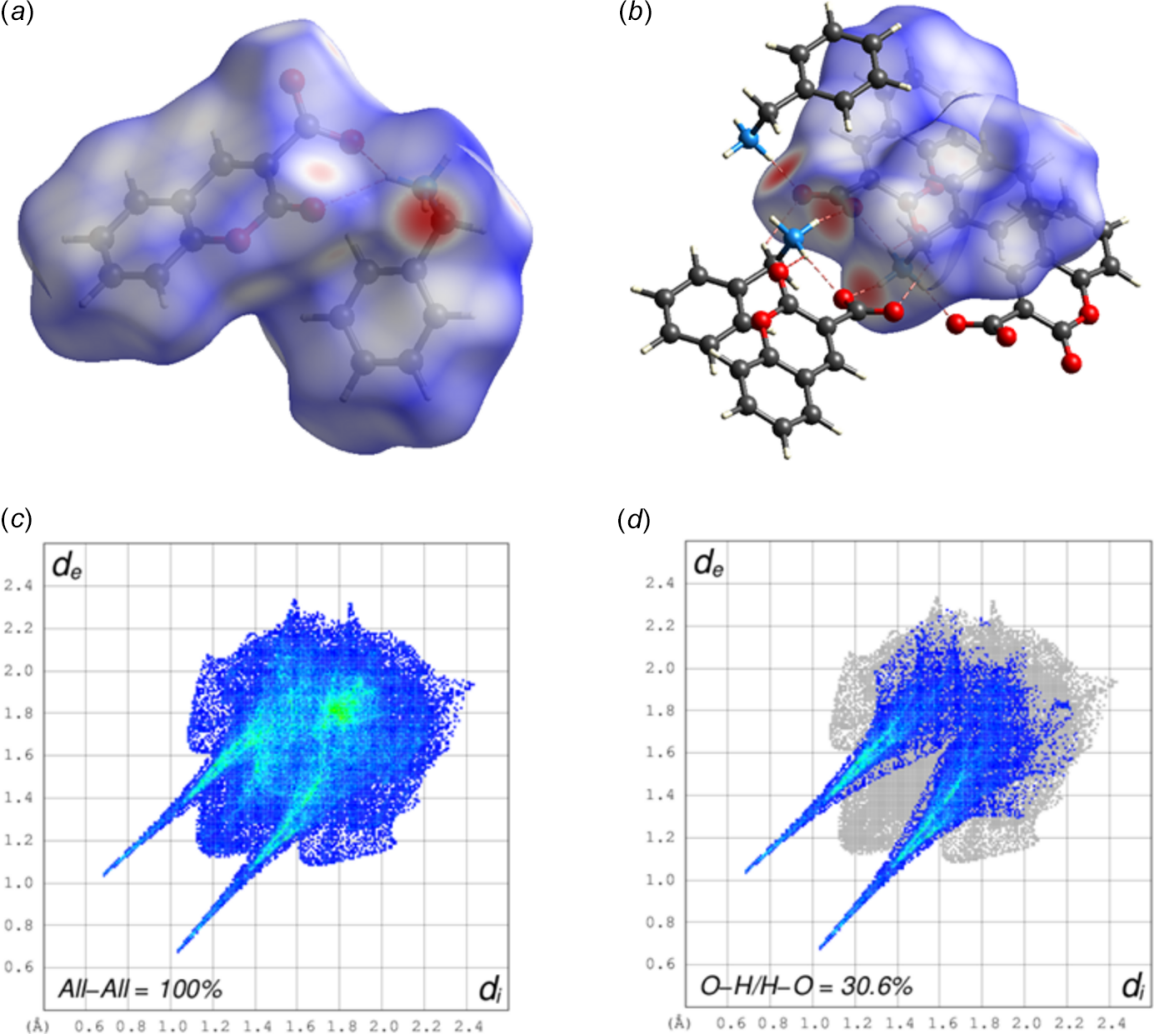
View of the Hirshfeld surface of (I) plotted over *d*_norm_ (*a*), the Hirshfeld surface with the N—H⋯O inter­molecular inter­actions (*b*),, two-dimensional fingerprint plots for the entire salt (*c*) and two-dimensional fingerprint plots showing sharp O⋯H/H⋯O spikes (30.6%) (*d*).

A Hirshfeld surface analysis (Hirshfeld, 1977 ▸; Spackman & Jayatilaka, 2009 ▸) was carried out using *Crystal Explorer 17.5* (Spackman *et al.*, 2021 ▸) to further quantify the inter­molecular inter­actions listed Table 1 ▸. The three-dimensional Hirshfeld surfaces plotted over *d*_norm_ separately for the 2-oxo-2*H*-chromene-3-carboxyl­ate moiety (*a*) and the phenyl­methanaminium fragment (*b*) are shown in Fig. 5 ▸. For both components of the salt, the inter­molecular inter­actions near the red spots are presented in Fig. 6 ▸. The two-dimensional fingerprint plots generated separately for the 2-oxo-2*H*-chromene-3-carboxyl­ate moiety (Fig. 7 ▸) and phenyl­methanaminium fragment (Fig. 8 ▸), show contributions from O⋯H/H⋯O, H⋯H, H⋯C/C⋯H, C⋯C, and O⋯C/C⋯O contacts of 44.3%, 23.6%, 19.1%, 11.4% and 1.2%, respectively, for the 2-oxo-2*H*-chromene-3-carboxyl­ate moiety, whereas 26.1%, 47.9%, 23.4%, 1.9% and 0.7%, respectively, for the phenyl­methanaminium fragment. Fig. 9 ▸*a* illustrates the Hirshfeld surface plotted over *d*_norm_ generated simultaneously for both fragments. The inter­molecular inter­actions present near the red spots are visualized in Fig. 9 ▸*b*. The 2D finger plot for the combined fragments (Fig. 9 ▸*c*) shows two sharp spikes at *d*_i_*+ d_e_* ≃ 1.7 Å resulting from O⋯H/H⋯O inter­actions with a contribution of 30.6% (Fig. 9 ▸*d*).

## 6. Anti­bacterial activity studies

The anti­bacterial activity of the title salt was evaluated by the agar disc-diffusion method following standard bioassay procedures (Atta-ur-Rahman *et al.*, 2001 ▸) and anti­microbial susceptibility testing guidelines recommended by the clinical and laboratory standards institute (Pierce *et al.*, 2023 ▸). The test organisms employed were *Staphylococcus aureus* (Gram-positive) and *Escherichia coli* (Gram-negative). The standard anti­bacterial drug ciprofloxacin was used as the reference control. The salt was dissolved in dimethyl sulfoxide (DMSO) to prepare a stock solution (1mg mL^−1^). Serial dilutions were prepared to obtain concentrations of 100, 75, 50, 40, 35, 30, 25, 20, 15, and 12.5 µg mL^−1^. A solvent control containing only DMSO was tested under identical experimental conditions to ensure that the solvent had no inhibitory effect on bacterial growth; no zone of inhibition was observed. Sterile filter paper discs (6 mm diameter) were impregnated with 10 µL of each test solution and dried under aseptic conditions. The discs were placed on Mueller–Hinton agar plates previously inoculated with standardized bacterial suspensions (∼10^8^ CFU mL^−1^; CFU = colony forming unit). The plates were incubated at 37 °C for 16–18 h. After incubation, the zones of inhibition were measured in millimetres (mm). The minimum inhibitory concentration (MIC) was determined as the lowest concentration showing visible inhibition of bacterial growth. The percentage inhibition of the title salt was calculated relative to the standard drug ciprofloxacin, whose inhibition zone was considered as 100%. The standard drug ciprofloxacin shows MIC values of 15 µg mL^−1^ against *S. aureus* and 15 µg mL^−1^ against *E. coli*. The title salt exhibited moderate anti­bacterial activity, with MIC values of 30 µg mL^−1^ against *S. aureus* and 25 µg mL^−1^ against *E. coli*, The slightly lower MIC against *E. coli* suggests that the title salt is marginally more effective against Gram-negative bacteria than Gram-positive bacteria.

## 7. Synthesis and crystallization

The title salt was prepared by dissolving 2-oxo-2*H*-chromene-3-carb­oxy­lic acid and phenyl­methanamine in a 1:1 molar ratio in a small excess of ethanol. The mixture was refluxed for 2 h to form a pale-yellow coloured solution. The mixture was cooled to room temperature, after which the solution was allowed to evaporate slowly to obtain crystals of the title salt. ATR-IR (*ν*_max_/cm^−1^): 2923 (N—H stretching of –NH^3+^), 1709 (C=O lactone stretching), 1570 (C=C aromatic ring stretching), 1320 (aromatic C—H stretching). ^1^H NMR (400 MHz, DMSO-*d*_6_, δ ppm): 4.52 (*s*, 2H, –CH_2_), 7.51–7.68 (*m*, 7H, Ar—H), 7.75–7.83 (*m*, 4H, Ar—H), 7.96–8.05 (*m*, 1H, Ar—H), 8.84 (*s*, 1H). ^13^C NMR (100 MHz, DMSO-*d*_6_, δ ppm): 146.6, 143.5, 140.1, 139.4, 138.5, 137.4, 133.9, 131.9, 129.4, 129.2, 128.4, 124.5, 122.5, 120.1, 120.0.

## 8. Refinement

Crystal data, data collection and structure refinement details are summarized in Table 2 ▸. H atoms were positioned with idealized geometry and refined using a riding model with N—H = 0.89 Å and *U*_iso_(H) = 1.2*U*_eq_(N), and C—H = 0.93 Å for CH groups, 0.97 Å for CH_2_ groups and U_iso_(H) = 1.2*U*_eq_(C).

## Supplementary Material

Crystal structure: contains datablock(s) I. DOI: 10.1107/S2056989026003403/vm2327sup1.cif

Structure factors: contains datablock(s) I. DOI: 10.1107/S2056989026003403/vm2327Isup2.hkl

Supporting information file. DOI: 10.1107/S2056989026003403/vm2327Isup3.cml

CCDC reference: 2542776

Additional supporting information:  crystallographic information; 3D view; checkCIF report

## Figures and Tables

**Table 1:** Hydrogen-bond geometry (Å, °) *Cg*4 is the centroid of the C11–C16 ring.

*D*—H⋯*A*	*D*—H	H⋯*A*	*D*⋯*A*	*D*—H⋯*A*
N1—H1*A*⋯O2	0.89	2.40	3.068 (3)	132
N1—H1*A*⋯O3	0.89	1.99	2.752 (3)	142
N1—H1*B*⋯O3^i^	0.89	1.94	2.791 (3)	158
N1—H1*C*⋯O4^ii^	0.89	1.83	2.716 (3)	174
C17—H17*B*⋯*Cg*4^iii^	0.97	2.76	3.613 (3)	147

Symmetry codes: (i) 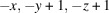; (ii) 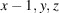; (iii) 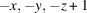.

**Table 2:** Experimental details

Crystal data	
Chemical formula	C_7_H_10_N^+^·C_10_H_5_O_4_^−^
*M*_r_	297.31
Crystal system, space group	Triclinic, *P* 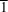
Temperature (K)	296
*a*, *b*, *c* (Å)	6.7505 (5), 7.7881 (6), 14.0809 (10)
α, β, γ (°)	79.644 (2), 84.583 (2), 74.960 (2)
*V* (Å^3^)	702.39 (9)
*Z*	2
Radiation type	Mo *K*α
μ (mm^−1^)	0.10
Crystal size (mm)	0.27 × 0.24 × 0.22

Data collection	
Diffractometer	Bruker SMART APEXII CCD
Absorption correction	Multi-scan (*SADABS*; Krause et al., 2015 ▸)
*T*_min_, *T*_max_	0.975, 0.979
No. of measured, independent and observed [*I* > 2σ(*I*)] reflections	21216, 3470, 3076
*R*_int_	0.043
(sin θ/λ)_max_ (Å^−1^)	0.668

Refinement	
*R*[*F*^2^ > 2σ(*F*^2^)], *wR*(*F*^2^), *S*	0.080, 0.161, 1.22
No. of reflections	3470
No. of parameters	200
H-atom treatment	H-atom parameters constrained
Δρ_max_, Δρ_min_ (e Å^−3^)	0.41, −0.38

Computer programs: *APEX2* and *SAINT* (Bruker, 2017 ▸), *SHELXT2018/3* (Sheldrick, 2015*a* ▸), *SHELXL2019/2* (Sheldrick, 2015*b* ▸), *Mercury* (Macrae *et al.*, 2020 ▸) and *publCIF* (Westrip, 2010 ▸).

## supplementary crystallographic information

### Phenylmethanaminium 2-oxo-2H-chromene-3-carboxylate Crystal data

**Table:**

C_7_H_10_N^+^·C_10_H_5_O_4_^−^	*Z* = 2
*M**_r_* = 297.31	*F*(000) = 312
Triclinic, *P*1	co-crystal
Hall symbol: -P 1	*D*_x_ = 1.406 Mg m^−^^3^
*a* = 6.7505 (5) Å	Mo *K*α radiation, λ = 0.71073 Å
*b* = 7.7881 (6) Å	Cell parameters from 3077 reflections
*c* = 14.0809 (10) Å	θ = 2–29°
α = 79.644 (2)°	µ = 0.10 mm^−^^1^
β = 84.583 (2)°	*T* = 296 K
γ = 74.960 (2)°	Prism, colourless
*V* = 702.39 (9) Å^3^	0.27 × 0.24 × 0.22 mm

### Phenylmethanaminium 2-oxo-2H-chromene-3-carboxylate Data collection

**Table:**

Bruker SMART APEXII CCD diffractometer	3470 independent reflections
Radiation source: fine-focus sealed tube	3076 reflections with *I* > 2σ(*I*)
Graphite monochromator	*R*_int_ = 0.043
Detector resolution: 1.97 pixels mm^-1^	θ_max_ = 28.4°, θ_min_ = 3.0°
φ and Ω scans	*h* = −8→9
Absorption correction: multi-scan (SADABS; Krause et al., 2015)	*k* = −10→10
*T*_min_ = 0.975, *T*_max_ = 0.979	*l* = −18→18
21216 measured reflections	

### Phenylmethanaminium 2-oxo-2H-chromene-3-carboxylate Refinement

**Table:**

Refinement on *F*^2^	Primary atom site location: structure-invariant direct methods
Least-squares matrix: full	Secondary atom site location: difference Fourier map
*R*[*F*^2^ > 2σ(*F*^2^)] = 0.080	Hydrogen site location: inferred from neighbouring sites
*wR*(*F*^2^) = 0.161	H-atom parameters constrained
*S* = 1.22	*w* = 1/[σ^2^(*F*_o_^2^) + (0.0372*P*)^2^ + 0.9952*P*] where *P* = (*F*_o_^2^ + 2*F*_c_^2^)/3
3470 reflections	(Δ/σ)_max_ < 0.001
200 parameters	Δρ_max_ = 0.41 e Å^−^^3^
0 restraints	Δρ_min_ = −0.38 e Å^−^^3^
0 constraints	

### Phenylmethanaminium 2-oxo-2H-chromene-3-carboxylate Special details

**Table:**

Geometry. All esds (except the esd in the dihedral angle between two l.s. planes) are estimated using the full covariance matrix. The cell esds are taken into account individually in the estimation of esds in distances, angles and torsion angles; correlations between esds in cell parameters are only used when they are defined by crystal symmetry. An approximate (isotropic) treatment of cell esds is used for estimating esds involving l.s. planes.

### Phenylmethanaminium 2-oxo-2H-chromene-3-carboxylate Fractional atomic coordinates and isotropic or equivalent isotropic displacement parameters (Å^2^)

**Table:**

	*x*	*y*	*z*	*U*_iso_*/*U*_eq_	
O1	0.1596 (2)	0.3659 (2)	0.10082 (12)	0.0219 (4)	
O2	−0.0292 (2)	0.4790 (2)	0.22176 (13)	0.0235 (4)	
O4	0.4445 (2)	0.5775 (2)	0.33560 (13)	0.0225 (4)	
O3	0.2113 (3)	0.4216 (2)	0.39393 (12)	0.0227 (4)	
C3	0.3466 (4)	0.3068 (3)	0.05274 (17)	0.0198 (5)	
C8	0.5288 (4)	0.3028 (3)	0.09229 (17)	0.0194 (5)	
C1	0.3342 (3)	0.4218 (3)	0.23032 (17)	0.0185 (5)	
C9	0.5163 (4)	0.3665 (3)	0.18286 (17)	0.0202 (5)	
H9	0.636260	0.369672	0.209257	0.024*	
N1	−0.2030 (3)	0.4531 (3)	0.43174 (14)	0.0181 (4)	
H1A	−0.093073	0.464509	0.393347	0.022*	
H1B	−0.216682	0.520552	0.477833	0.022*	
H1C	−0.314408	0.489019	0.397146	0.022*	
C10	0.3279 (3)	0.4804 (3)	0.32791 (17)	0.0186 (5)	
C7	0.7141 (4)	0.2416 (3)	0.04013 (18)	0.0238 (5)	
H7	0.838499	0.236871	0.065044	0.029*	
C4	0.3414 (4)	0.2571 (3)	−0.03637 (18)	0.0251 (5)	
H4	0.217204	0.263651	−0.062042	0.030*	
C2	0.1425 (3)	0.4249 (3)	0.18859 (16)	0.0177 (5)	
C5	0.5256 (4)	0.1976 (4)	−0.08612 (18)	0.0280 (6)	
H5	0.525708	0.163133	−0.146024	0.034*	
C12	−0.3402 (4)	0.0714 (3)	0.39657 (19)	0.0278 (6)	
H12	−0.460831	0.109011	0.433133	0.033*	
C11	−0.1712 (4)	0.1384 (3)	0.40422 (17)	0.0216 (5)	
C16	0.0057 (4)	0.0840 (3)	0.34773 (18)	0.0248 (5)	
H16	0.118753	0.129561	0.351336	0.030*	
C14	−0.1528 (5)	−0.1060 (4)	0.2794 (2)	0.0342 (7)	
H14	−0.145768	−0.188100	0.237969	0.041*	
C17	−0.1777 (4)	0.2615 (3)	0.47659 (18)	0.0268 (6)	
H17A	−0.290682	0.252259	0.523598	0.032*	
H17B	−0.051546	0.221327	0.510838	0.032*	
C13	−0.3301 (5)	−0.0512 (4)	0.3348 (2)	0.0337 (6)	
H13	−0.443188	−0.096414	0.330775	0.040*	
C6	0.7118 (4)	0.1886 (4)	−0.04749 (19)	0.0268 (5)	
H6	0.834902	0.146506	−0.081327	0.032*	
C15	0.0152 (4)	−0.0376 (4)	0.28602 (19)	0.0307 (6)	
H15	0.134884	−0.073852	0.248626	0.037*	

### Phenylmethanaminium 2-oxo-2H-chromene-3-carboxylate Atomic displacement parameters (Å^2^)

**Table:**

	*U*^11^	*U*^22^	*U*^33^	*U*^12^	*U*^13^	*U*^23^
O1	0.0168 (8)	0.0306 (9)	0.0216 (9)	−0.0079 (7)	−0.0042 (6)	−0.0082 (7)
O2	0.0143 (8)	0.0301 (9)	0.0261 (9)	−0.0030 (7)	−0.0025 (7)	−0.0075 (7)
O4	0.0149 (8)	0.0278 (9)	0.0288 (9)	−0.0063 (7)	−0.0049 (7)	−0.0116 (7)
O3	0.0173 (8)	0.0328 (9)	0.0207 (9)	−0.0074 (7)	−0.0012 (6)	−0.0098 (7)
C3	0.0197 (11)	0.0202 (11)	0.0208 (11)	−0.0068 (9)	−0.0022 (9)	−0.0033 (9)
C8	0.0188 (11)	0.0207 (11)	0.0207 (11)	−0.0069 (9)	−0.0033 (9)	−0.0045 (9)
C1	0.0167 (11)	0.0211 (11)	0.0195 (11)	−0.0063 (9)	−0.0048 (9)	−0.0035 (9)
C9	0.0175 (11)	0.0245 (11)	0.0212 (12)	−0.0072 (9)	−0.0060 (9)	−0.0051 (9)
N1	0.0117 (9)	0.0227 (10)	0.0212 (10)	−0.0027 (7)	−0.0044 (7)	−0.0073 (8)
C10	0.0125 (10)	0.0220 (11)	0.0218 (11)	−0.0002 (8)	−0.0066 (8)	−0.0075 (9)
C7	0.0175 (11)	0.0296 (13)	0.0257 (13)	−0.0061 (9)	−0.0019 (9)	−0.0073 (10)
C4	0.0253 (13)	0.0322 (13)	0.0213 (12)	−0.0107 (10)	−0.0061 (10)	−0.0059 (10)
C2	0.0164 (11)	0.0200 (11)	0.0184 (11)	−0.0063 (9)	−0.0039 (8)	−0.0032 (9)
C5	0.0349 (14)	0.0346 (14)	0.0182 (12)	−0.0114 (11)	−0.0009 (10)	−0.0100 (10)
C12	0.0268 (13)	0.0245 (12)	0.0281 (14)	−0.0038 (10)	0.0011 (10)	0.0009 (10)
C11	0.0297 (13)	0.0167 (11)	0.0168 (11)	−0.0023 (9)	−0.0012 (9)	−0.0032 (9)
C16	0.0266 (13)	0.0250 (12)	0.0223 (12)	−0.0047 (10)	−0.0022 (10)	−0.0042 (10)
C14	0.0532 (18)	0.0216 (12)	0.0283 (14)	−0.0036 (12)	−0.0131 (13)	−0.0080 (11)
C17	0.0380 (15)	0.0203 (12)	0.0203 (12)	−0.0020 (10)	−0.0030 (10)	−0.0054 (9)
C13	0.0354 (15)	0.0308 (14)	0.0386 (16)	−0.0138 (12)	−0.0152 (12)	0.0003 (12)
C6	0.0248 (13)	0.0305 (13)	0.0255 (13)	−0.0074 (10)	0.0052 (10)	−0.0082 (10)
C15	0.0340 (15)	0.0288 (13)	0.0236 (13)	0.0043 (11)	−0.0028 (11)	−0.0066 (11)

### Phenylmethanaminium 2-oxo-2H-chromene-3-carboxylate Geometric parameters (Å, º)

**Table:**

O1—C2	1.379 (3)	C4—C5	1.380 (4)
O1—C3	1.383 (3)	C4—H4	0.9300
O2—C2	1.206 (3)	C5—C6	1.397 (4)
O4—C10	1.249 (3)	C5—H5	0.9300
O3—C10	1.261 (3)	C12—C13	1.387 (4)
C3—C4	1.384 (3)	C12—C11	1.393 (4)
C3—C8	1.389 (3)	C12—H12	0.9300
C8—C7	1.405 (3)	C11—C16	1.385 (4)
C8—C9	1.437 (3)	C11—C17	1.510 (3)
C1—C9	1.348 (3)	C16—C15	1.382 (4)
C1—C2	1.463 (3)	C16—H16	0.9300
C1—C10	1.517 (3)	C14—C13	1.379 (4)
C9—H9	0.9300	C14—C15	1.389 (4)
N1—C17	1.484 (3)	C14—H14	0.9300
N1—H1A	0.8900	C17—H17A	0.9700
N1—H1B	0.8900	C17—H17B	0.9700
N1—H1C	0.8900	C13—H13	0.9300
C7—C6	1.372 (4)	C6—H6	0.9300
C7—H7	0.9300	C15—H15	0.9300
			
C2—O1—C3	122.79 (17)	O2—C2—C1	126.6 (2)
O1—C3—C4	116.9 (2)	O1—C2—C1	116.8 (2)
O1—C3—C8	120.6 (2)	C4—C5—C6	120.7 (2)
C4—C3—C8	122.5 (2)	C4—C5—H5	119.6
C3—C8—C7	118.1 (2)	C6—C5—H5	119.6
C3—C8—C9	118.0 (2)	C13—C12—C11	120.5 (3)
C7—C8—C9	123.9 (2)	C13—C12—H12	119.8
C9—C1—C2	120.5 (2)	C11—C12—H12	119.8
C9—C1—C10	119.8 (2)	C16—C11—C12	119.0 (2)
C2—C1—C10	119.8 (2)	C16—C11—C17	120.6 (2)
C1—C9—C8	121.3 (2)	C12—C11—C17	120.3 (2)
C1—C9—H9	119.3	C15—C16—C11	120.3 (2)
C8—C9—H9	119.3	C15—C16—H16	119.8
C17—N1—H1A	109.5	C11—C16—H16	119.8
C17—N1—H1B	109.5	C13—C14—C15	119.5 (3)
H1A—N1—H1B	109.5	C13—C14—H14	120.3
C17—N1—H1C	109.5	C15—C14—H14	120.3
H1A—N1—H1C	109.5	N1—C17—C11	113.3 (2)
H1B—N1—H1C	109.5	N1—C17—H17A	108.9
O4—C10—O3	126.4 (2)	C11—C17—H17A	108.9
O4—C10—O3	126.4 (2)	N1—C17—H17B	108.9
O4—C10—C1	116.4 (2)	C11—C17—H17B	108.9
O3—C10—C1	117.1 (2)	H17A—C17—H17B	107.7
O3—C10—C1	117.1 (2)	C14—C13—C12	120.1 (3)
C6—C7—C8	120.1 (2)	C14—C13—H13	119.9
C6—C7—H7	119.9	C12—C13—H13	119.9
C8—C7—H7	119.9	C7—C6—C5	120.3 (2)
C5—C4—C3	118.2 (2)	C7—C6—H6	119.8
C5—C4—H4	120.9	C5—C6—H6	119.8
C3—C4—H4	120.9	C16—C15—C14	120.5 (3)
O2—C2—O1	116.62 (19)	C16—C15—H15	119.7
O2—C2—O1	116.62 (19)	C14—C15—H15	119.7
O2—C2—C1	126.6 (2)		
			
C2—O1—C3—C4	−177.8 (2)	C3—O1—C2—C1	−0.3 (3)
O1—C3—C8—C7	180.0 (2)	C9—C1—C2—O2	−176.4 (2)
C4—C3—C8—C7	−1.6 (4)	C10—C1—C2—O2	3.9 (4)
O1—C3—C8—C9	−1.7 (3)	C9—C1—C2—O2	−176.4 (2)
C4—C3—C8—C9	176.7 (2)	C10—C1—C2—O2	3.9 (4)
C2—C1—C9—C8	−2.4 (4)	C9—C1—C2—O1	1.2 (3)
C10—C1—C9—C8	177.3 (2)	C10—C1—C2—O1	−178.5 (2)
C3—C8—C9—C1	2.6 (3)	C3—C4—C5—C6	−0.2 (4)
C7—C8—C9—C1	−179.2 (2)	C13—C12—C11—C16	1.4 (4)
C9—C1—C10—O4	42.9 (3)	C13—C12—C11—C17	−175.7 (2)
C2—C1—C10—O4	−137.4 (2)	C12—C11—C16—C15	−1.2 (4)
C9—C1—C10—O3	−134.8 (2)	C17—C11—C16—C15	175.9 (2)
C2—C1—C10—O3	44.9 (3)	C16—C11—C17—N1	78.0 (3)
C9—C1—C10—O3	−134.8 (2)	C12—C11—C17—N1	−104.9 (3)
C2—C1—C10—O3	44.9 (3)	C15—C14—C13—C12	0.0 (4)
C3—C8—C7—C6	0.3 (4)	C11—C12—C13—C14	−0.8 (4)
C9—C8—C7—C6	−177.8 (2)	C8—C7—C6—C5	0.9 (4)
O1—C3—C4—C5	180.0 (2)	C4—C5—C6—C7	−1.0 (4)
C8—C3—C4—C5	1.5 (4)	C11—C16—C15—C14	0.4 (4)
C3—O1—C2—O2	177.5 (2)	C13—C14—C15—C16	0.2 (4)
C3—O1—C2—O2	177.5 (2)		

### Phenylmethanaminium 2-oxo-2H-chromene-3-carboxylate Hydrogen-bond geometry (Å, º)

*Cg*4 is the centroid of the C11–C16 ring.

**Table:**

*D*—H···*A*	*D*—H	H···*A*	*D*···*A*	*D*—H···*A*
N1—H1*A*···O2	0.89	2.40	3.068 (3)	132
N1—H1*A*···O3	0.89	1.99	2.752 (3)	142
N1—H1*B*···O3^i^	0.89	1.94	2.791 (3)	158
N1—H1*C*···O4^ii^	0.89	1.83	2.716 (3)	174
C17—H17*B*···*Cg*4^iii^	0.97	2.76	3.613 (3)	147

Symmetry codes: (i) −*x*, −*y*+1, −*z*+1; (ii) *x*−1, *y*, *z*; (iii) −*x*, −*y*, −*z*+1.
